# Single-Lobe Living Donor Liver Transplant in a Morbidly Obese Cirrhotic Patient Preceded by Laparoscopic Sleeve Gastrectomy

**DOI:** 10.1155/2013/279651

**Published:** 2013-12-10

**Authors:** Sunil Taneja, Subash Gupta, Manav Wadhawan, Neerav Goyal

**Affiliations:** ^1^Department of Gastroenterology and Hepatology, Indraprastha Apollo Hospital, Delhi 110076, India; ^2^Department of Surgical Gastroenterology & Liver Transplantation, Centre for Liver & Biliary Surgery, Indraprastha Apollo Hospital, Delhi 110076, India

## Abstract

Nonalcoholic steatohepatitis (NASH) is a stage of nonalcoholic fatty liver disease (NAFLD), and, in most patients, it is associated with obesity and metabolic syndrome with progression to end-stage liver disease in about 20% of patients (McCullough (2004); Matteoni et al. (1999); Liou and Kowdley (2006)). It has been estimated that between 20 and 30% of patients with end-stage cirrhosis referred for liver transplantation (LT) evaluation and 30 to 70% of LT recipients exhibit some degree of obesity (Muñoz and ElGenaidi (2005)). Management of obesity in chronic liver disease patients is not only difficult but also preludes them from undergoing major bariatric surgery due to associated high morbidity and mortality. Here, we present a case report of a morbidly obese patient who underwent laparoscopic sleeve gastrectomy followed by single-lobe living donor liver transplantation (LDLT) with a successful outcome. We believe that this is the first report of successful LDLT following planned weight loss to facilitate LDLT.

## 1. Case Report

A 29-year-old young male law graduate, morbidly obese since adolescence, has been symptomatic since the last 7 years with fatigue, generalized weakness, and fluctuating jaundice. He was 160 kg at the time of presentation with a BMI of 55.36 kg/m^2^. He was diagnosed to have cirrhosis with persistently high bilirubin which fluctuated between 2 and 5 mg/dL. His Child's score at presentation was B9 with a MELD score of 14. Extensive evaluation for the etiology of liver disease including viral markers and autoimmune markers and iron and copper studies were all negative. There was no history of alcohol intake; hence, in view of morbid obesity and dyslipidemia, the diagnosis of nonalcoholic steatohepatitis for cirrhosis was considered. His gastroduodenoscopy showed small varices with portal hypertensive gastropathy, and a triple-phase CT scan of the abdomen showed a shrunken liver with splenomegaly, multiple portosystemic collaterals, and no ascites. He was listed for deceased donor liver transplantation and advised weight reduction with dietary and behavioral measures. However, he did not show any significant improvement over the next three months. Since he was morbidly obese and had failed medical therapy for weight reduction, he was planned for bariatric surgery after counseling for worsening of liver disease in the postoperative period and a possible requirement for immediate liver transplantation in that eventuality. A prospective living donor was identified in the family and completely worked up in case the patient showed signs of worsening liver disease following bariatric surgery. He underwent laparoscopic sleeve gastrectomy and had a smooth postoperative course. Following bariatric surgery, he had a significant reduction of weight from 160 kg to 102 kg in six-month period. However he developed worsening of his liver disease in the form of progressive jaundice, ascites, and recurrent episodes of hepatic encephalopathy. His Child's score worsened from B9 to C12, and MELD score increased to 24 over the next six months. In the absence of availability of cadaveric donor, living donor liver transplantation was considered as his weight had now decreased substantially. He underwent a modified right-lobe LDLT from his sister-in-law as the donor with a graft weight of 837 grams and an acceptable GRBWR of 0.81. The postoperative period was smooth except for basal atelectasis, which required intense physiotherapy and intermittent BIPAP for a few days.

## 2. Discussion

Liver transplant in obese patients has been associated with increased morbidity and mortality in the postoperative period [1, 2]. Options for weight reduction for obese patients in need of liver transplant include a noninvasive approach of rigorous dietary and behavioral modification employed both before and after transplantation. However, this noninvasive approach may not be successful for all patients, particularly those with long-standing severe obesity. Bariatric surgery may be suitable for patients with early-stage liver disease [3], but it is not indicated for patients with decompensated liver disease [4]. Aggressive noninvasive pretransplant weight loss protocol was unsuccessful in attaining weight loss in this patient. Hence, considering the relatively compensated liver disease and young age of the patient, a decision for laparoscopic sleeve gastrectomy was undertaken to make him a suitable candidate for liver transplantation. Sleeve gastrectomy was chosen over gastric bypass due to decreased technical complexity and the lack of malabsorption, which may impact early posttransplant immunosuppression levels. With the lack of cadaveric organs, LDLT was considered as his weight had decreased substantially.

Inadequate GRBWR is a problem encountered in obese patients. Cadaveric transplant is ideal; or else, dual-lobe transplant would need to be considered in societies where cadaveric donation is uncommon. Dual-graft living donor liver transplantation can overcome small-for-size syndrome in large recipients and ensure donor safety by leaving sufficient remnant liver volume in the donor. However, dual-lobe transplant is often not possible and is also technically demanding. This patient had a successful outcome by a planned, preoperative bariatric surgery and single-right-lobe graft. To the best of our knowledge, this is the first report of successful bariatric surgery for weight loss and planned living-donor-related liver transplantation in a morbidly obese patient. However, we suggest that such technically demanding procedures should be undertaken only in large-volume centers with adequate surgical expertise and critical care management.

## 3. Conclusion

Bariatric surgery in end-stage liver disease is risky and may be associated with poor outcome. However, in certain special circumstances and centers with backup for living donor liver transplantation, such surgeries may be considered in improving the outcome of morbidly obese cirrhotic patients.
